# What do we know about the language extinction rate?

**DOI:** 10.1017/ext.2026.10019

**Published:** 2026-07-30

**Authors:** Michaël Boissonneault, Harald Hammarström

**Affiliations:** 1 Département de démographie et des sciences de la population, Université de Montréal, Montréal, QC, Canada; 2 Department of Linguistics and Philology, Uppsala Universitet, Uppsala, Sweden

**Keywords:** language extinction, language endangerment, intergenerational transmission, linguistic diversity, extinction rates

## Abstract

Does one language really disappear every two weeks? The statistic is widely cited in discussions of language endangerment, yet its empirical basis remains unclear. This article traces the origins of the claim and reviews the evidence on which it rests. We first revisit the conjecture that half of the world’s languages were already no longer transmitted to the next generation in the early 1990s and examine how this assumption gave rise to the “one language every two weeks” figure. Using assessments from major linguistic catalogues, including the UNESCO Atlas and Ethnologue, we show that the proportion of languages no longer transmitted to younger generations is substantially lower than originally assumed. We then review more recent attempts to estimate the rate of language extinction from documented disappearances, rather than from current intergenerational transmission statuses. These studies suggest a much lower rate of roughly one language every six to thirteen weeks but rely on methods that are subject to important – yet difficult to quantify – sources of bias. We conclude that the widely cited claim that one language disappears every two weeks is probably too high, while the accuracy of more recent estimates cannot presently be determined. Drawing on approaches developed in biological extinction research, we conclude by outlining how methods that account for the population at risk, supported by data documenting the demographic trajectories of the world’s languages, could enable more robust estimates of the current rate of language extinction.

## Impact statement

Claims that languages are disappearing at an alarming pace – often summarized by the widely cited statement that “one language dies every two weeks” – have played a major role in shaping public awareness, academic debates and policy discussions on linguistic diversity. This article shows that this figure is not a direct observation of current language loss, but rather a reinterpretation of a conjecture made more than three decades ago on the basis of limited data. By revisiting both the origins of this claim and the evidence now available, the study provides a more accurate understanding of what is currently known about the rate of language disappearance. The findings have important implications beyond academic research. For policymakers, educators and organizations involved in language documentation and revitalization, they highlight the need to base decisions on robust and transparent evidence rather than on widely circulated but poorly understood statistics. For the broader public, the study contributes to a more informed understanding of language loss – one that recognizes both the seriousness of the issue and the uncertainties that still surround it. More generally, the article demonstrates how improved data and methods can transform our understanding of global challenges. By drawing parallels with approaches used in biodiversity research, it also opens new avenues for measuring language extinction more accurately. This contributes to ongoing international efforts, such as the International Decade of Indigenous Languages, by clarifying what is currently known – and what remains to be measured – about the future of the world’s linguistic diversity.

“Every two weeks, one language disappears.” For more than twenty-five years, this claim has circulated widely in popular science writing, policy documents, academic publications and media campaigns. It has appeared in statements from UNESCO, been repeated in major newspapers such as *The Guardian* and *The New York Times* (Wilford, 2007; McVeigh, 2023) and has been widely publicized by organizations such as the National Geographic Society (Strochlic, 2018). Through constant repetition across such venues, the phrase has taken on the status of an unquestioned fact, described by some scholars as “a fundamental truth” about the pace at which languages are disappearing (Belew and Simpson, 2018b, p. 44).

Despite its widespread use, the evidential basis of the “two-weeks” claim is questionable. Recent studies describe it as a “vague extrapolation” or “a far cry” from what current data allow us to infer, suggesting instead that a language disappears every six or even thirteen weeks (Belew and Simpson, 2018b, p. 44; Simons, 2019, p. 11). These revised numbers have already begun to influence reference works. For instance, the 2024 MIT Press volume *Endangered Languages* states that careful estimates suggest that one language stops being used every three months, rather than every two weeks (Adamou, 2024). The BBC program *More or Less* recently devoted an entire episode to the topic, stating, “We don’t think that number is right” (BBC Radio, 2024, 00:01:25). Yet, these newer estimates have not gained universal acceptance, as many linguists report field experiences that, to them, feel closer to the older figure. Rather than resolving the matter, the result has been a widening gap between lived experience and available quantitative evidence.

The aim of this review is to clarify the origin of the “one language every two weeks” statistic and to examine whether current data support it. Previous work has reviewed parallels between language and species endangerment (Bromham, 2023) or mathematical approaches to competition between languages spoken in the same area (Boissonneault and Vogt, 2021). In contrast, this review focuses specifically on the pace at which languages are disappearing, a dimension of language vitality that has received less systematic attention despite its growing importance in both theoretical and policy discussions, including those associated with the ongoing UNESCO International Decade of Indigenous Languages (UNESCO, 2021).

This review is structured in three parts. The first examines the origin of the “one language every two weeks” statistic. It shows that it derives from a conjecture put forward in 1992 about the proportion of languages no longer being transmitted to the next generation and that it was originally intended to concern the future of linguistic diversity rather than the current pace of extinction. The second part reassesses the evidence on intergenerational transmission using the data now available and shows that the share of languages no longer being learned by children remains far below what was originally assumed. The third part turns to recent work that seeks to estimate the rate of language extinction directly from observed disappearances rather than from transmission status. We show that this approach suffers from limitations that are likely to bias estimates downward. As a result, neither the original estimate nor the more recent ones appear to accurately reflect the current rate. Finally, the discussion considers how methods that account for the population at risk, supported by data documenting the demographic trajectories of the world’s languages, could enable more robust estimates in the future.

Because this review concerns the rate at which languages disappear, a brief note on terminology is warranted. The term *language extinction* has been questioned by some Indigenous scholars and communities, who instead prefer expressions such as *dormant*, *sleeping*, or *reawakening* for languages with no remaining first-language speakers (Leonard, 2008; Perley, 2012). Where appropriate, we therefore avoid the term language extinction in favour of alternative expressions, while retaining it when discussing previous studies or the concept of an extinction rate.

## “3,000 of 6,000”

The widespread belief that one language disappears every two weeks can be traced back to David Crystal’s book *Language Death*, published in 2000 (Crystal, 2000). In discussing how many languages are endangered and why it is difficult to produce a single figure, Crystal refers to an earlier claim by Michael Krauss. In an essay published eight years earlier, Krauss had written that 3,000 languages could stop being spoken during the twenty-first century given their current intergenerational transmission status (i.e., whether the language is still being passed from parents to children) (Krauss, 1992). “To meet that time frame,” Crystal writes, “one language must die, on average, every two weeks or so” (Crystal, 2000, p. 19).

As we can see – and according to Crystal himself – Crystal’s statement was not meant as a measurement of the present situation, but as a way of conveying the implications of Krauss’s projection if it were evenly distributed over a century (Lamble, 2024). Nevertheless, as the phrase circulated in the media and academic literature, this context was often forgotten, and the illustrative calculation was gradually transformed into a statistic about the current pace at which languages stop being spoken.

Krauss’s prediction that half of the world’s languages could disappear by 2,100, meanwhile, became another foundational reference point for discussions of language endangerment – less often taken up in popular publications but more in academic ones (Abrams and Strogatz, 2003; Bromham et al., 2021). As such, it is worth documenting further the context in which it was formulated.

Krauss supports the claim that half of the world’s languages could disappear by 2,100 with two distinct lines of reasoning. First, Krauss proceeds to compile rough counts of “moribund” languages – defined as languages no longer being transmitted to children – for regions familiar to him or to colleagues with whom he had discussed the issue. These regions include Alaska and the “Soviet North,” Canada and the United States, Mesoamerica, South America, Australia, and parts of Russia. Across these areas, the regional estimates reported by Krauss sum to 624 of 1,252 languages – almost exactly 50% – that are no longer being transmitted (Krauss, 1992; pp. 4–5).

Krauss acknowledges that these regions represent only a fraction of the world’s linguistic diversity, as entire continents (including Africa, most of Eurasia, and the Pacific) are omitted. To assess the global situation, he therefore turns to *Ethnologue.* First published in 1951 to guide Bible translation efforts, *Ethnologue* had by that time appeared in several editions and had become the principal catalogue of the world’s languages (Pittman, 1951; Erard, 2005; Hammarström, 2015). Drawing on information from the latest edition available to him (the 12th, published in 1988), Krauss notes that roughly half of the world’s languages were still considered viable enough to justify Bible translation efforts, while the “condition of about 40% [was] inadequately known, and 10% [were] classed as ‘nearly extinct’ or ‘highly bilingual’, not warranting translation work” (Krauss, 1992; p. 6).

Krauss’s discussion nevertheless raises two important questions. First, it is not entirely clear how the Ethnologue categories relate to his definition of moribund languages. Second, the fact that the vitality of 40% of the world’s languages was unknown complicates any attempt at a global assessment. If one assumes that languages classified as “nearly extinct” or “highly bilingual” correspond to Krauss’s definition of moribund, and that the 40% of languages with unknown status resembled the 60% for which information was available, this would imply that about 17% of the world’s languages were moribund. Krauss instead suggests that although a majority of the languages with unknown status were probably still viable, “as many as 20%” of the world’s languages might already be moribund (Krauss, 1992; p. 6).

Krauss’s regional estimates thus suggested that roughly half of the languages in the areas he considered were moribund, whereas the global evidence on which he relied pointed to a substantially lower figure, one that he suggested could be as high as 20%. Krauss ultimately resolved this discrepancy by appealing to informal expert judgment:

However, two other linguists with wide experience have both independently guessed, along with me, that the total may be more like 50%, or at least that the number of languages which, at the rate things are going, will become extinct during the coming century is 3,000 of 6,000 (p. 6).

Thus, despite the most comprehensive resource available at the time suggesting a markedly lower proportion, Krauss adopts 50% as his estimate.

Whether Krauss’s decision was based on a dismissal of the *Ethnologue* statistics or a deliberate inflation of the numbers to convey a sense of urgency is not clear. Ironically, however, his estimate that half of the world’s languages might disappear was soon treated as conservative in later discussions (Crystal, 2000; Thomason, 2015). The reason lies in what follows immediately after the 50% estimate in Krauss’s essay. In the next paragraph, Krauss introduces an even starker scenario based on counts of languages with official status and large speaker populations (set at 100,000 or more). These are the languages he considers “safe” in the long run, and he argues that they represent only about 10% of the world’s languages. Under this reasoning, the remaining 90% would ultimately face either “death” or “doom,” meaning that they would cease to be learned by children at some point in the 21st or 22nd century (p. 7). Although Krauss presents this as a longer-term projection rather than as a description of the current situation, the distinction was often lost in later discussions, contributing to the widespread perception that even the 50% estimate was conservative.

In sum, despite its great subsequent influence, Krauss’s projection was never based on comprehensive global data, but rather on selective regional evidence, incomplete *Ethnologue* figures, and informal expert judgment. Although it originated from estimates of languages thought to no longer be transmitted to children, it was widely interpreted as a prediction of how many languages would disappear in the future. Through Crystal’s later calculation, it also became the ultimate source of the “one language every two weeks” statistic, whose influence ultimately extended far beyond Krauss’s original projection.

## The world’s languages in crisis?

Krauss’s projection that half of the world’s languages were already moribund in the early 1990s was formulated at a time when the global linguistic record was still highly incomplete. Today, the documentation of the world’s languages is far more complete (Seifart et al., 2018), and for the vast majority of languages some form of vitality assessment now exists. Given the importance Krauss’s prediction has acquired in discussions of language vitality worldwide, it is worth examining how it compares with the evidence now available.

Several catalogues now provide global assessments of language endangerment. Of these, only the UNESCO Atlas and Ethnologue systematically distinguish between languages that are still being transmitted to children and those that are not. They are therefore the focus of this section because they allow us to assess Krauss’s claim that half of the world’s languages would no longer be transmitted to children by the end of the twenty-first century. Other catalogues are discussed later where relevant, but more briefly because they do not classify languages according to intergenerational transmission.

The development of the UNESCO Atlas followed soon after the publication of Krauss’s “The World’s Languages in Crisis.” Growing concern about language endangerment during the 1980s and 1990s led UNESCO to establish a working group on endangered languages in 1993 (Robins and Uhlenbeck, 1991; Crystal, 2000). One outcome was the UNESCO *Red Book of Endangered Languages*, which led to the publication of the first edition of the *Atlas of the World’s Languages in Danger* in 1996 (Wurm, 1996). A second edition followed in 2001, and a substantially revised and expanded third edition was published in 2010 (Wurm, 2001; Moseley, 2010). Unlike the first two editions, which covered only a subset of endangered languages, the 2010 edition aimed to include all languages whose intergenerational transmission was at least partially interrupted.

UNESCO later launched the *World Atlas of Languages* in 2019 to document all languages regardless of vitality, but the platform has been offline for maintenance since at least 2025 (UNESCO, 2025). We therefore rely on the 2010 *Atlas of the World’s Languages in Danger* for statistics on intergenerational transmission and refer to it hereafter as the UNESCO Atlas. In that resource, 1,663 of the 2,471 languages listed fall into the categories “Definitely endangered,” “Severely endangered,” or “Critically endangered,” which, according to UNESCO’s definition, indicate that intergenerational transmission has been completely interrupted (Table 1). The 2010 Atlas does not specify the total number of living languages worldwide, making it impossible to calculate the proportion represented by these 1,663 languages directly. Relating them instead to the 6,909 languages listed in the 2009 edition of *Ethnologue*, published closest in time to the Atlas, yields an estimate of roughly 24% of the world’s languages. These figures suggest that the proportion of moribund languages may be only about half of what Krauss estimated in 1992.

**Table 1.** Comparison of the EGIDS and UNESCO language vitality scales, highlighting categories indicating a complete break in intergenerational transmission Table 1. long description.

Category label		Category description	
EGIDS	UNESCO scale	Youngest speakers	Intergenerational transmission
0 (International) through 6a (Vigorous)	Safe	Children	Ongoing
6b (Threatened)	Vulnerable	Children	Partially interrupted
7 (Shifting)	Definitely endangered	Childbearing age adults	Completely interrupted
8a (Moribund)	Severely endangered	Grandparents	Completely interrupted
8b (Nearly Extinct)	Critically endangered	Elderly/grandparents	Completely interrupted
9 (Dormant) and 10 (Extinct)	Extinct	There are no speakers left	

Ethnologue began assigning languages to vitality categories in its 17th edition (2014) using the Expanded Graded Intergenerational Disruption Scale (EGIDS) (Lewis et al., 2014). The EGIDS combines the UNESCO framework’s finer distinctions among endangered languages with Fishman’s Graded Intergenerational Disruption Scale (GIDS), which classifies different stages of language shift among languages that are still being transmitted (Fishman, 1991; Lewis and Simons, 2010) (Table 1). Since it distinguishes between languages that are still being transmitted and those that are not, it provides a second source of evidence for assessing Krauss’s prediction.

In the 17th edition, 1,192 languages were classified in the categories “Shifting,” “Moribund,” and “Nearly Extinct” (Lewis et al., 2014). These are the three EGIDS levels indicating a complete break in intergenerational transmission, corresponding broadly to UNESCO’s “Definitely endangered,” “Severely endangered,” and “Critically endangered” categories. Since this edition listed 7,106 languages in total, this corresponds to roughly 17% of the world’s languages (Table 2).

**Table 2.** Comparison of different estimates regarding the current number of languages not being transmitted and the language extinction rate Table 2. long description.

	Krauss/Crystal	UNESCO Atlas	Ethnologue/Simons	ELP
Not transmitted	50%	24%	16–17%	–
Actual extinctions	26 / year	–	9 / year	4 / year

*Note:* The Krauss/Crystal estimates are based on Krauss’ prediction (1992) and Crystal’s reinterpretation thereof (2000). The UNESCO Atlas estimates are based on UNESCO’s classification presented in Moseley (2010) (UNESCO Atlas). The Ethnologue/Simons estimates are from Lewis et al. (2014), Eberhard et al. (2025) and Simons (2019). The ELP estimates are from Belew and Simpson (2018b), Campbell et al. (2013) and Campbell and Okura (2018).

Later editions make it possible to examine whether this picture has changed. In the 28th edition (published in 2025), 1,164 languages fall into the same three categories (Eberhard et al., 2025). This slight decrease does not imply that the situation has improved but largely reflects the fact that languages previously classified in these categories have since become dormant. The total number of known living languages nonetheless increased slightly to 7,159, and the proportion classified as moribund decreased by about half a percentage point to around 16%. In either case, the Ethnologue tally is considerably lower than the proportion suggested by the UNESCO Atlas and only about one-third of Krauss’s estimate.

It is worth mentioning, however, that many languages are assigned by Ethnologue to the adjacent EGIDS category “6b (Threatened).” These include languages with partially interrupted transmission, and their number has increased substantially between the 17th and the 28th editions. While this suggests that some may soon move into the fully interrupted group, its proportion will nonetheless remain far below the 50% implied by Krauss.

The remaining catalogues frequently consulted for endangerment statistics either do not provide direct information on intergenerational transmission or do so for only a subset of the world’s languages. For instance, the *Catalogue of Endangered Languages* (ELCat) provides detailed assessments of more than 3,000 endangered languages through the ELP website (Endangered Languages Project, 2025). These assessments are based on the *Language Endangerment Index* (LEI), a composite scale that includes intergenerational transmission as one of its four components (Lee and Van Way, 2016). However, transmission data are missing for many entries (Hauk and Heaton, 2018), making it impossible to derive a reliable count of languages corresponding to Krauss’s “moribund” category.

Another catalogue is *Glottolog*, which since 2018 has been assigning languages to one of six endangerment levels through its Agglomerated Endangerment Status (Hammarström et al., 2018). However, nearly all of *Glottolog*’s vitality assessments come from the ELP, the UNESCO Atlas or *Ethnologue.* As a result, *Glottolog* does not provide additional evidence for evaluating Krauss’s prediction.

Finally, the recently created *LinguaMeta* project compiles speaker numbers and other language metadata from multiple sources (Ritchie et al., 2024). However, it aggregates existing information rather than providing independent assessments of language vitality and was developed chiefly to support multilingual natural language processing rather than language endangerment research.

Taken together, the two catalogues that allow a direct assessment – the UNESCO Atlas and *Ethnologue* – suggest that between roughly eleven hundred and sixteen hundred languages currently lack intergenerational transmission. In terms of the proportion of all languages, this corresponds to between 16% and 24% of the world’s languages. These figures are therefore between one-third and one-half of what Krauss proposed. Based on current evidence, the claim that half of the world’s languages are on the verge of disappearing within the present century appears difficult to reconcile with currently available vitality assessments.

## “A more realistic approach”?

In the previous section, we reviewed the evidence for Krauss’s prediction that half the world’s languages would stop being spoken before the end of the current century, which later became the basis of the “one language every two weeks” statistic. Since its publication, many have interpreted this statement in a literal sense, as referring to the present pace at which languages disappear. Following this reasoning, one way to assess its accuracy is to examine how many languages are believed to have ceased being spoken in recent decades.

The ELP, Ethnologue and the UNESCO Atlas all include categories for languages with no living speakers – variously termed “extinct,” “dormant,” or “reawakening.” These classifications follow specific conventions, including a cutoff year used to ensure that catalogues include all languages known to have been spoken after that date. Some also provide information about when a language’s last speaker died, either as specific years (e.g., 1995) or broader time ranges (e.g., the 1990s).

One approach that has been proposed to estimate the rate of language extinction is to divide the number of languages classified as extinct or dormant by the period over which those disappearances are believed to have occurred. The result is an average number of extinctions per year – or, expressed differently, the average number of days, weeks or months between extinctions. This has been described as a “more realistic approach to calculating current extinction rates” as it relies on observed language disappearances rather than indirect indicators of endangerment (Belew and Simpson, 2018b, p. 44).

This approach has been pursued independently by two groups of authors, both of whom used their estimates to assess Crystal’s statistic. The first group comprises researchers associated with the ELP, who presented their findings in several publications, including chapters in the edited volume *Cataloguing the World’s Endangered Languages* (Campbell and Belew, 2018). Focusing on extinctions believed to have occurred since 1960, the authors identified between 230 and 256 languages that have become dormant, depending on the source (Belew et al., 2018; Campbell and Okura, 2018; Belew and Simpson, 2018b). Dividing these totals by the period of observation – roughly 56 to 57 years in their analyses – they obtained an average of about 4.1 to 4.3 extinctions per year. This corresponds to roughly one language disappearing every three months, leading authors to conclude that the claim that a language disappears every two weeks is “not supported by the evidence” (Belew and Simpson, 2018b, p. 43).

A similar approach was taken in 2019 by Gary Simons of SIL Global (Simons, 2019). His analysis relies on revised assessments of whether languages are still being spoken published in the 22nd edition of *Ethnologue* (Eberhard et al., 2019), which provides more systematic information about when last speakers may have died. Simons estimates the mean number of languages becoming dormant or extinct during successive 25-year periods extending back to the late eighteenth century. For the periods 1971–1995 and 1996–2018 – those most relevant for assessing Crystal’s statistic – he finds an average of slightly more than nine languages per year. Simons describes this result as “a far cry from the widely cited ‘one language dies every 14 days’” (p. 11).

Despite differing from one another by roughly a factor of two, the recently published estimates reviewed above are all substantially lower than the widely cited claim that one language disappears every two weeks (Table 2). Some of the authors who have produced them have also criticized the continued circulation of the “one language every two weeks” statistic, calling for restraint in the use of what they describe as “extreme estimates” (Belew and Simpson, 2018b, p. 44). We offer in the remainder of this section a new perspective on these recently published estimates.

**Figure 1.**
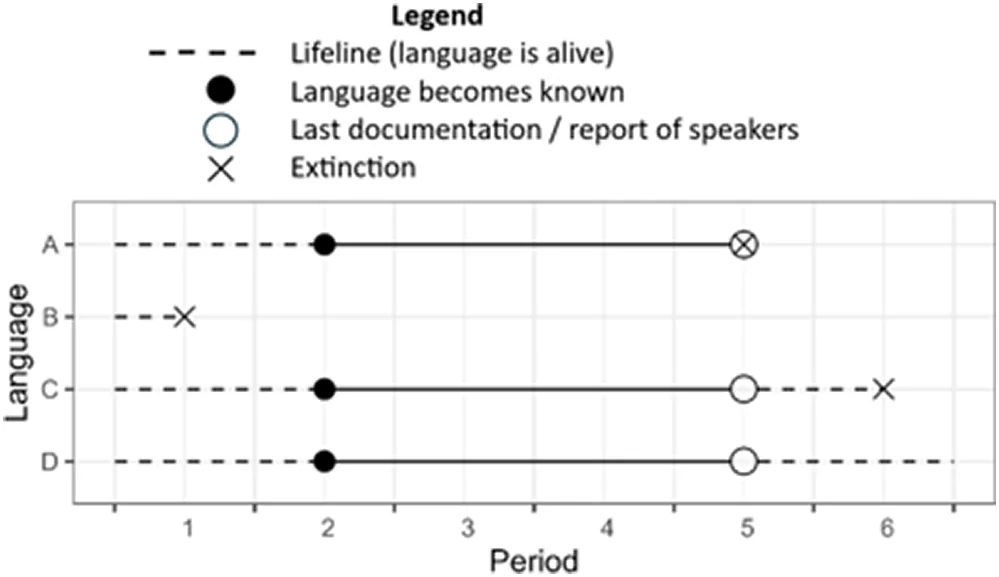
Illustration of four possible trajectories linking language disappearance and its observation. Four hypothetical languages are followed over six time periods. All are still spoken in period 1 but become known to researchers only from period 2 onward. Language A ceases to be spoken in period 5, and this is documented. Language B ceases to be spoken in period 1, before being documented, and is therefore never recorded. Language C is observed until period 5 but ceases to be spoken in period 6 without this being reported, so its disappearance remains undocumented. Language D is observed until period 5 and continues to be spoken (the most common outcome). In this example, three languages cease to be spoken, but only one disappearance is documented. Figure 1. long description.

**Figure 2.**
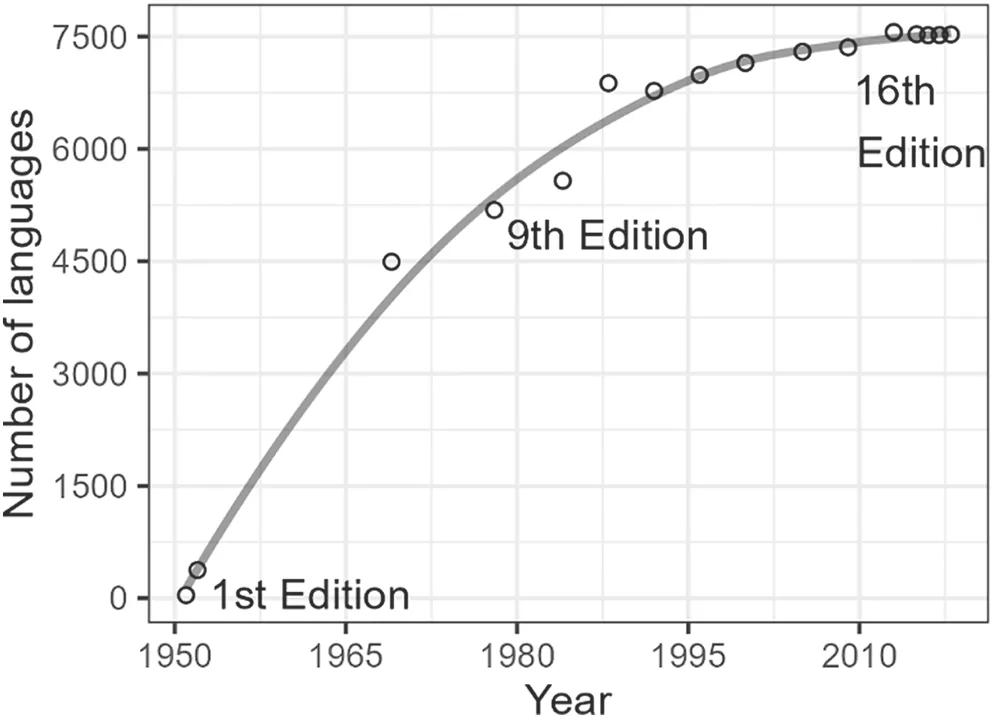
Number of languages inventoried in selected editions of Ethnologue. The dots represent the actual numbers, the line an interpolation. The actual numbers include both living and dormant or extinct languages. Figure 2. long description.

There are two reasons to be sceptical of approaches that estimate the language extinction rate solely from observed disappearances (Figure 1). The first is that the approach taken by the ELP group and by Simons counts only disappearances of languages previously known to exist. To assess the extent of this issue, it is useful to consider how the number of documented languages has evolved over time. In Ethnologue, for example, only forty languages were listed in the first edition in 1951 (Pittman, 1951). This number increased to around four thousand in the 1970s and roughly six thousand in the late 1980s, before stabilizing in the 2010s at around seven thousand (Figure 2).

These figures should not be interpreted as a complete record of the world’s linguistic diversity at each point in time. The earliest editions of *Ethnologue*, in particular, did not aim to provide a comprehensive inventory of the world’s languages, and consequently omitted many languages that were already known to linguists (Hammarström et al., 2024). Many of these languages were only minimally documented, however, making it difficult to determine whether or when they ceased to be spoken. Moreover, some languages undoubtedly disappeared before they were documented at all. Such languages can never be counted as dormant, because a language cannot be recorded as having disappeared unless it is first known to have existed. Although the number of such cases is impossible to estimate, they become increasingly likely the further one goes back in time. Extinction counts extending back to the 1960s and 1970s, when the historical record was much less complete, therefore almost certainly underestimate the true number of disappearances.

A second limitation concerns more recent extinctions that may not yet be reflected in catalogue data. Languages listed as living in catalogues such as *Ethnologue* or the ELP are often classified based on sources that date from the 1990s or early 2000s (Table 3). Moreover, these dates usually refer to the publication of a source rather than to the year when the underlying fieldwork was conducted. As a result, languages can remain classified as living even though they may in fact have gone extinct years earlier, especially when the last assessment is old and reports only a handful of speakers. Because these approaches rely on languages already classified as dormant or extinct, such cases introduce an additional source of downward bias into the estimation procedure.

**Table 3.** Distribution of languages by the reference year of speaker number estimates Table 3. long description.

Period	Number of languages	Percentage
2020–2025	1,325	18.4
2010–2019	2,025	28.1
2000–2009	2,521	35.0
1990–1999	771	10.7
1980–1989	419	5.8
Pre–1980	138	1.9
Total	7,199	100

*Source*: Ethnologue, 29th edition (Eberhard et al., 2026).

*Note:* Numbers represent languages with at least one population estimate that includes a reference year. For languages listed in multiple countries, the earliest available reference year is used (e.g., a language is counted in the 2020–2025 category if its earliest population estimate dates from that period).

Evidence of this problem can be found in the ELP data. In one chapter of *Cataloguing the World’s Endangered Languages*, Hauk and Heaton (2018) list 191 languages reported to have ten speakers or fewer according to sources dating from 2006 or earlier. They note that many of these languages may already have become dormant, yet they were nonetheless excluded from the counts used to derive estimates of about four extinctions per year.

In contrast, many of the languages that ELP classifies as living are classified as extinct by Ethnologue. Ethnologue appears somewhat more inclined to assign languages with outdated information to the dormant category, even in the absence of explicit reports of their disappearance. This may help explain part of the fact that Simons estimated more than twice the rate of the ELP group using Ethnologue data. However, such reclassifications are not applied systematically and may correct some of the limitations discussed above while introducing others. Their overall effect on the resulting estimates is therefore difficult to assess.

These limitations also make it difficult to draw conclusions about how the pace of language extinction may have changed over time. Extinction counts will tend to be biased downward in earlier periods, when fewer languages had been documented, but also in more recent ones, because the disappearance of languages is often recorded with considerable delay. Apparent trends in the data may therefore reflect changes in documentation and reporting practices rather than real changes in the underlying rate of language loss. Yet some authors have attempted to detect such trends. Using ELCat records, Belew and Simpson (2018a) suggest “a rapid increase” in the number of extinctions over the 1960–2017 period (although, interestingly, Simons’ analysis points instead to a roughly stable rate between 1971–1995 and 1996–2018) (Belew and Simpson, 2018a, p. 55). Given the uncertainties discussed above, however, we do not believe that temporal trends can be inferred from raw counts alone.

Taken together, these limitations imply that estimates of the language extinction rate derived from catalogue data cannot be interpreted as direct estimates of the true rate of language disappearance. Although sources of bias can be identified, their overall effect on the resulting estimates remains unknown. Likewise, the current data and methods do not allow reliable conclusions about whether the pace of language loss is increasing, decreasing or stable over time.

## Towards more reliable estimates of language extinction

Important advances over the past decades have greatly improved our knowledge of the world’s linguistic diversity and of the processes that threaten it. Linguistic catalogues are far more complete than they were fifty years ago. Systems for classifying language vitality, such as the EGIDS and the LEI, have also been developed and widely adopted. Categories such as threatened, endangered or critically endangered are now routinely used to describe whether languages are still being transmitted to younger generations. In addition, more information is now available on language speaker numbers and, in some cases, on the deaths of last speakers. Taken together, these developments provide a much clearer picture than was available when early warnings about language extinction were formulated.

Recent studies have also taken an important step by attempting to estimate the extinction rate from observed disappearances rather than from indirect indicators such as the proportion of endangered languages (Belew et al., 2018; Campbell and Okura, 2018; Belew and Simpson, 2018b; Simons, 2019). By counting languages whose last speakers are believed to have died within a given period, these approaches rely on more concrete evidence of language loss. However, the procedure used to convert these observations into an extinction rate remains limited, as it consists of dividing the number of recorded extinctions by the period during which they are believed to have occurred. This calculation implicitly assumes both that the inventory of known languages was reasonably complete throughout the period considered and that language disappearances are documented without substantial delay. As we have seen, neither assumption is fully satisfied. Estimates based on extinction counts should therefore be interpreted cautiously, as the methods used to derive them are affected by biases whose overall effect remains uncertain.

None of these limitations implies that the currently available data are useless. Similar challenges have long been encountered in other fields, and useful methodological approaches have been developed to address them. A central principle is that extinction counts should be related to the population at risk. In biology, for example, only a fraction of the world’s species have been described, and even fewer have reliable assessments of their conservation status (Bland et al., 2015). Yet extinction rates are routinely estimated by relating observed extinctions to the number of species known to exist over time, commonly expressed as extinctions per million species-years (Pimm et al., 1995, 2014). Comparable approaches could, in principle, be applied to languages. Statistical methods such as imputation may also help account for languages that have disappeared but whose disappearance has not yet been documented (Van Buuren and Groothuis-Oudshoorn, 2011). Although these approaches necessarily rely on assumptions that are difficult to verify, they provide a promising starting point for developing more robust estimates.

Before such methods can be applied, however, the available data must be carefully compiled and evaluated. At present, the main catalogues of the world’s languages differ substantially in the type and quality of information they provide. Ethnologue has progressively improved its practice of citing sources, and many speaker numbers and extinction dates can now be traced to identifiable references. At the same time, it does not provide sources for endangerment status, and information from previous editions is not preserved in a way that allows researchers to reconstruct the historical lifelines of languages. Much of its detailed information is also now available only through subscription (Matacic, 2020), making large-scale independent analyses more difficult. The ELP provides greater transparency by systematically listing sources and often comparing information from multiple references. However, many of these references are themselves drawn from Ethnologue, the UNESCO Atlas, or secondary compilations, resulting in considerable redundancy. Glottolog, by contrast, is highly transparent in its classification practices but does not provide information on speaker numbers. These differences illustrate that no single catalogue currently provides all the information needed to study language extinction rates in a consistent way. Developing a database that combines these features will require careful evaluation of existing information, identifying original sources and distinguishing reliable evidence from repeated or uncertain claims.

Historical reconstruction alone will not be sufficient. Improving the collection of new information in the field would also greatly strengthen future research. Linguistic fieldwork understandably focuses primarily on documenting the structure and use of languages (Austin, 2016). Nevertheless, systematically recording basic demographic information would greatly improve efforts to understand and quantify language loss. Information on speaker numbers, age structure, and the proportion of community members who still use the language would support more reliable assessments of language vitality and help estimate the status of languages for which recent data are lacking. Even when documenting an entire speech community is impractical, recording the age and sex of participants in linguistic fieldwork could provide valuable insights into patterns of intergenerational transmission.

What, then, can now confidently be said about the rate at which languages are disappearing? Recent attempts to estimate the current rate represent an important methodological advance, as they rely on observed language disappearances rather than indirect indicators of endangerment. However, as our review has shown, the methods currently used are affected by biases whose overall effect on the resulting estimates remains unknown. It is therefore not yet possible to determine with confidence how closely the available estimates reflect the true rate of language disappearance. At the same time, the widely cited claim that one language dies every two weeks is also difficult to reconcile with the available evidence. That figure ultimately rests on the assumption that about half of the world’s languages are no longer being transmitted to younger generations, whereas current estimates place this proportion closer to 16% to 24 %. Moreover, as our review has shown, the “one language every two weeks” statistic was never originally intended as a direct description of the present rate of language extinction, but rather emerged as a reinterpretation of a prognosis formulated more than three decades ago on the basis of much more limited data.

Some authors have warned against the proliferation of extreme estimates, arguing that claims that 90 % of the world’s languages are doomed risk trivializing the issue of language loss (Belew and Simpson, 2018b). While such criticisms are justified when estimates rely on speculation or repeated citations without empirical support, the lower figures – sometimes presented as corrective (Adamou, 2024) – should also be interpreted carefully. At present, the available evidence does not allow the rate of language extinction to be determined with precision. Further progress will depend on improving the demographic documentation of languages, the transparent organization of well-structured databases, and the methods used to analyze the available data.

## Data Availability

There is no data associated with this manuscript.

## Long descriptions

### Table 1. Long description

The table consists of four columns: Category label E G I D S, Category label UNESCO scale, Youngest speakers, and Intergenerational transmission.

* Row 1: E G I D S 0 International through 6a Vigorous corresponds to UNESCO Safe. Youngest speakers are children and transmission is ongoing.

* Row 2: E G I D S 6b Threatened corresponds to UNESCO Vulnerable. Youngest speakers are children and transmission is partially interrupted.

* Row 3: E G I D S 7 Shifting corresponds to UNESCO Definitely endangered. Youngest speakers are childbearing adults and transmission is completely interrupted.

* Row 4: E G I D S 8a Moribund corresponds to UNESCO Severely endangered. Youngest speakers are grandparents and transmission is completely interrupted.

* Row 5: E G I D S 8b Nearly Extinct corresponds to UNESCO Critically endangered. Youngest speakers are elderly or grandparents and transmission is completely interrupted.

* Row 6: E G I D S 9 Dormant and 10 Extinct correspond to UNESCO Extinct. There are no speakers left.

Navigate back to Table 1..

### Table 2. Long description

The table consists of five columns and three rows.

Column headers from left to right are:

1. An empty cell for row labels.

2. Krauss forward slash Crystal.

3. U N E S C O Atlas.

4. Ethnologue forward slash Simons.

5. E L P.

Row 1, Not transmitted:

- Krauss forward slash Crystal: 50 percent.

- U N E S C O Atlas: 24 percent.

- Ethnologue forward slash Simons: 16 to 17 percent.

- E L P: No data indicated by a dash.

Row 2, Actual extinctions:

- Krauss forward slash Crystal: 26 per year.

- U N E S C O Atlas: No data indicated by a dash.

- Ethnologue forward slash Simons: 9 per year.

- E L P: 4 per year.

Navigate back to Table 2..

### Figure 1. Long description

A horizontal timeline graph with the Y axis labeled Language (A, B, C, D) and the X axis labeled Period (1 through 6).

Legend at the top defines four symbols:

* Dashed line: Lifeline (language is alive).

* Solid black circle: Language becomes known.

* Open circle: Last documentation or report of speakers.

* X: Extinction.

Data flow for each language:

* Language A: Dashed line from period 1 to 2. At period 2, a black circle marks it becoming known, followed by a solid line. At period 5, an X inside an open circle indicates documented extinction.

* Language B: Dashed line starts at period 1 but is immediately interrupted by an X before period 2. No other symbols appear, representing unrecorded extinction.

* Language C: Dashed line from period 1 to 2. Black circle at period 2 followed by a solid line. Open circle at period 5 marks last documentation. A dashed line continues to period 6, where an X marks undocumented extinction.

* Language D: Dashed line from period 1 to 2. Black circle at period 2 followed by a solid line. Open circle at period 5 marks last documentation. A dashed line continues past period 6, indicating the language remains alive.

Navigate back to Figure 1..

### Figure 2. Long description

The horizontal X axis is labeled Year, ranging from 1950 to 2010 in 15 year increments. The vertical Y axis is labeled Number of languages, ranging from 0 to 7500 in increments of 1500.

Data points and trends:

* The graph begins near the origin in 1951 with a point labeled 1st Edition, showing nearly 0 languages.

* A steep linear increase occurs between 1951 and 1980. A data point near 1980 is labeled 9th Edition, positioned at approximately 5200 languages.

* The growth rate slows after 1980, forming an asymptotic curve that levels off toward the top right.

* A final cluster of data points near 2010 is labeled 16th Edition, reaching a peak of approximately 7500 languages.

* An interpolated solid gray line connects the general path of the circular data points, which are scattered slightly above and below the line during the middle years.

Navigate back to Figure 2..

### Table 3. Long description

The table consists of three columns: Period, Number of languages, and Percentage.

* Period 2020 to 2025: 1,325 languages, 18.4 percent.

* Period 2010 to 2019: 2,025 languages, 28.1 percent.

* Period 2000 to 2009: 2,521 languages, 35.0 percent.

* Period 1990 to 1999: 771 languages, 10.7 percent.

* Period 1980 to 1989: 419 languages, 5.8 percent.

* Pre 1980: 138 languages, 1.9 percent.

* Total: 7,199 languages, 100 percent.

The data indicates that the majority of language speaker estimates (35 percent) date from the period between 2000 and 2009.

Navigate back to Table 3..
