# Supplementary material for: What do we know about the language extinction rate?
Source: Camb Prism Extinct. 2026 Jul 30;4:e14. doi: 10.1017/ext.2026.10019 (PMC13748987; doi:10.1017/ext.2026.10019)
Supplement: Peer review history

# Reviewer report: Review: What do we know about the language extinction rate? — R0/PR2

**DOI:** 10.1017/ext.2026.10019.pr2

Review of “What do we know about the language extinction rate?” for Cambridge Prism. I have grouped my comments into Major, Medium and Minor indicating weight.

I really like this paper and I think it is very needed. I believe that there are a couple of additions and tweaks that will make the paper even more brilliant, nothing too dramatic. The paper is sound and has a great structure.

Major

I think it’s worthwhile to include a discussion, however short, on the politics of the labels “extinct”, “dormant” and “reawakening”. Languages are different kinds of entities from biological species, it can feel very harsh to talk about “extinction” as if it was a non-emotional issue.

Linked to this, it would be good to include some discussion on the health impacts of language loss. Possible citations include:

* Durie, M., Milroy, H., & Hunter, E. (2009). Mental health and the Indigenous peoples of Australia and New Zealand. In L. J. Kirmayer & G. G. Valaskakis (Eds.), Healing traditions: The mental health of Aboriginal peoples in Canada (pp. 36–55). UBC Press.

* Evans, N. (2022). Words of wonder: Endangered languages and what they tell us (2nd ed.). Wiley-Blackwell.

* Auger, M. D. (2016). Cultural continuity as a determinant of Indigenous peoples’ health: A meta-synthesis of qualitative research in Canada and the United States. International Indigenous Policy Journal, 7(3).

* Whalen, D. H., Lewis, M. E., Gillson, S., McBeath, B., Alexander, B., & Nyhan, K. (2022). Health effects of Indigenous language use and revitalization: A realist review. International Journal for Equity in Health, 21(1), 169.

We are in the UNESCO International Decade of Indigenous Languages. It wouldn’t go amiss to mention this fact and some basic descriptions of community work at language reawakening and maintenance. Possible citations include:

* Zobule, A. G., & McDougall, D. (2026). Grammar as a bridge: Empowering Indigenous learners in a Solomon Islands language school.

* Woods, L. (2025). Reawakening Wangaaypuwan and A Plain Language Grammar.

* Woods, L (2023) Something’s Gotta Change Redefining Collaborative Linguistic Research. ANU Press

* Gaby, A., & Woods, L. (2020). Toward linguistic justice for Indigenous people: A response to Charity Hudley, Mallinson, and Bucholtz. Language, 96(4), e268-e280

* Lewis, M. P. (2023). Sustaining Language Use: Bridging the gap between language communities and linguists. In Endangered Languages in the 21st Century (pp. 105-118). Routledge.

* Jesse Hodgetts (2024) Ngiyampaa ngiya guthigu ngiyali (Ngiyampaa Words for Talking About Songs): Creating Mayi (Aboriginal) Language for Musicology in a Language Revitalization Context, Musicology Australia, 46:1, 47-66, DOI: 10.1080/08145857.2024.2390194

* Plumb, M. H., Dubcovsky, A., Guzmán, M. G., Lillehaugen, B. D., & Lopez, F. H. (2024). Growing a bigger linguistics through a Zapotec agenda. Anne H. Charity Hudley, Christine Mallinson & Mary Bucholtz (eds.), Decolonizing linguistics, 363-381.

* Bobongie-Harris, F., O’Brien, G., Priddle, C., Bobongie, F., & Fiaalii, S. (2026). Country, community, and indigenous languages–A framework used in an early learning centre in Queensland to develop understandings around first nations languages. Australasian Journal of Early Childhood, 51(1), 33-47.

Line 361: There is a new resource of language populations (Ritchie et al 2024), from the Google research team. Unlike Ethnologue, it’s not behind a paywall and has sources for each data-point. Full ref: Ritchie, S., van Esch, D., Okonkwo, U., Vashishth, S., & Drummond, E. (2024, May). LinguaMeta: Unified metadata for thousands of languages. In Proceedings of the 2024 Joint International Conference on Computational Linguistics, Language Resources and Evaluation (LREC-COLING 2024) (pp. 10530-10538). Data: https://github.com/google-research/url-nlp/tree/main/linguameta

Medium

It would be good to state explicitly that population size is not necessarily a good marker of endangerment. Some languages have always been pretty “small”, there’s a reason most experts focus on transmission to younger generation instead. This is obvious to many linguists, but maybe not all other readers.

Transform newer estimates to “x languages in 2 weeks”

I think that in order for the message to really hit home, the authors need to translate the new ranges into “x languages per 2 weeks”, using partially the same assumptions as Krauss and Chrystal (i.e. target year is 2100, there’s approx 6-7,000 languages). I feel conflicted suggesting this, because I think there’s the possibility that this will be misinterpreted the same way that Chrystal and Krauss have already been. However, I think currently it is possible to a reader to walk away and later mainly register "that statistic isn’t right“ but not have a straightforward comparison to scale the newer numbers to. The paper will be more impactful if the authors translate into this scale than if they say ”proportion is lower“ as is done in the abstract. Given all the caveats in the paper, it would of course be necessary to give a range rather than a point estimate. However, if we are to ”deprogram" linguists of this notion, I think this is a necessary step. This should happen in the abstract, in table 1 and in end of discussion.

Besides lack of transparency, I think it is also worthwhile to mention that Ethnologue has also been behind a paywall for many (most?) users since 2016 - with exceptions for users from low-income countries and contributors.

Page 212: Glottolog’s AES actually does, in some instances, come from Glottolog itself! As odd as that might sound, it seems that AES is being transformed from a purely aggregated metric from other sources to a hybrid of a resource managed by Glottolog. For example, ngub1239’s AES status is sourced to “Glottolog”: https://github.com/glottolog/glottolog/blob/master/languoids/tree/atla1278/volt1241/benu1247/bant1294/sout3152/narr1281/cent2260/west2968/nzad1235/lwer1234/ding1244/loan1238/klce1234/kiko1235/nucl1804/kiko1234/kamb1321/kila1239/sout3249/west2874/vili1240/lumb1251/ngub1240/ngub1239/md.ini

In the paper describing AES (Hammarström et al 2018, Simultaneous Visualization of Language Endangerment

and Language Description) they describe Glottolog as an input source for AES with the values “extinct” ~ “not extinct”, but there appears to have been changes since then since ngub1239’s status is “shifting”.

Line 411: Glottolog contains the parameters Last and first year of documentation. While not the same as requested “last observed to be spoken”, these two parameters may still be of use in this enterprise. This data is recorded for approximately 1,000 languoids in Glottolog.

Minor

Lines 55 and 56: is the reference here to the BBC radio episode? If so and if the journal style permits, just use author & year instead of title & year.

Line 71 81: I take it that the page references are meant to refer to pages inside Krauss 1992. If that is correct and the journal style permits, please use the full format (e.g. Krauss 1992:5-6).

Place table 1 after the mention of “ELP” in the text.

# Reviewer report: Review: What do we know about the language extinction rate? — R0/PR3

**DOI:** 10.1017/ext.2026.10019.pr3

**Authors:** Gary Simons

I commend the authors for an answer to the question posed in the title that is both well-researched and well-written. I believe that it will be ready for publication after some minor revisions. My feedback below is in two halves. The first half is meant to engage with the authors in a discussion of their final conclusions about what it will take to know the language extinction rate more precisely. These are not points where I would necessarily expect to see revisions, but are simply meant to offer ideas. The second half of the feedback gives comments on points of detail that probably do warrant a revision.

I have no problem agreeing with the conclusion that, “The estimates [of language extinction rates] reviewed in this article are almost certainly lower than the true rate, since they count only extinctions that have been confirmed or recorded in the available catalogues.” But given the spread between 4 extinctions per year and 9 extinctions per year for the two rates that are calculated from “available catalogues,” this summary statement leaves much to be desired. Since it says that both values are “almost certainly low,” then they are saying that they believe 9 is low, which means that they believe 4 to be off by more than a factor of 2. But how far off is 9? By just a few percent, or by another factor of 2? It is unfortunate that the conclusion does not give the reader any help in making a judgement as to what the actual rate might be, especially given the context set in the opening sentence of the article which identifies an extinction rate of 26 as being the one that is the most widely quoted (even among linguists). Rather than leaving the reader hanging with the impression that the rate of 9 is way off, it would be more satisfying if the authors could give some consideration to the “effect sizes” of languages missing from the inventory and of languages in the inventory failing to yet be recorded as extinct.

Given the large mismatch between 4 and 9, I feel that a higher priority than the remedies suggested in the closing sentence of the article would be to call for a third-party review of the existing data. The ELCat has a list of “between 230 and 256 languages that have become dormant or extinct” since 1960 (depending on which source one follows). For the same period of time, Ethnologue has a list of exactly 534 languages (since it makes an editorial judgement in the case of conflicting witnesses). With such a large gap in results for two analyses which purport to be calculating the same thing, it is clear that something is wrong. Our discipline would be well served by a third party review of both lists. The “mark and recapture” technique from wildlife biology (https://en.wikipedia.org/wiki/Mark_and_recapture) could be applied. We essentially have two projects that have gone into the wild and tagged all the members they could find of a species that has an unknown population (namely, the number of languages that have gone silent since 1960). Given the number of animals tagged by the first expedition, the number of animals tagged by the second expedition, and the number that were tagged in common by both, biologists have formulas for estimating the total population of the species. A method like that might be able to give us a measure of how much lower these lists are than the number of languages that have actually gone dormant. By studying those two datasets it might also be possible to start quantifying the effect size of the latency in the population data for the languages we already know about. That is, what is an estimate for the proportion of languages recorded as critically endangered that are in fact already dormant? While we may not be able to know which specific languages have the wrong vitality status, we may be able to calculate a proportion that probably have the wrong status and then apply that as a correction factor to the final calculation of the extinction rate.

An even better way to estimate the effect size for the number of actual languages that have yet to be recorded in the inventory of known languages would be to do a mark and recapture analysis of Glottolog versus Ethnologue. Given that both make use of ISO 639, the analysis as to which languages they have in common has already been performed. This could provide an estimate of the proportion by which the Ethnologue inventory of languages is lower than the actual number of languages, which could in turn be proposed as the proportion by which the estimate of the language extinction rate based on Ethnologue data is low.

I also have doubts as to whether “an approach analogous to the use of extinctions per million species-years” will be as useful to linguistics as it is to biology. I can think of three key differences between the two fields:

(1) We are trying to estimate the rate of extinction for just one thing, languages, while biology is dealing with a huge number of classes of things: e.g., every taxonomic class, order, or family. Because these differ so widely (e.g. the number of species of mammals versus beetles), comparing extinctions per class has little meaning. Therefore, extinctions per million species-years is used to standardize across classes with grossly different sizes. We don’t need that when dealing with only one class.

(2) We are primarily trying to estimate the rate of extinction at the present (and also looking at how the rate has been changing over recent generations), while biologists are trying to establish background extinction rates over the eons and to document mass extinction events that have occurred in geologic time. The fossil record makes it possible to observe extinctions that occurred millions of years ago and observed extinctions per million species-years gives a way to standardize extinction across different periods of time that have different lengths and different numbers of species observed in the fossil record. For languages, we can’t really do something that is comparable since our analog to fossils would be writing, but the vast majority of languages throughout history have left no written records.

(3) Another problem that standardizing to extinctions per million species-years solves for biology is that of not knowing how many species are in a given class. On the surface this sounds comparable to the situation for languages, but the problem for biology is orders of magnitude larger. The inventory of large mammals is essentially complete, so that extinctions per year can actually be calculated for that class. But the class of insects, for instance, is another story altogether. Gemini tells us that approximately 1 million species have been identified, while biologists estimate that the actual number of species could be anywhere from 5 million to 30 million. Thus there is no way to extrapolate from observed extinctions per year to an estimate of actual extinctions per year for the whole class of insects. Standardizing to observed extinctions per million-species-years solves that problem. But we don’t really have that problem for languages. As Figure 2 in the paper shows, the inventory of known languages is reaching an asymptote, and so it can be treated as a complete class (with a fractional correction for the number of missing members that is estimated from a technique like mark and capture as suggested above).

That concludes the discussion of points concerning the problem of knowing the language extinction rate more precisely. The remaining comments deal with details that probably do call for revision.

L80ff: “the Ethnologue figures … indicated that 10% of the 60% of languages for which vitality information was available were ‘nearly extinct’ (that is, roughly 17% of all languages)”

This is describing how Krauss interpreted the 1988 edition of Ethnologue, but it didn’t sound right to me. For instance, I knew that the 2000 edition (which is the first one I prepared for online publication) listed only 417 languages as nearly extinct (https://www.ethnologue.com/14/nearly_extinct/). That is only 6% of the total number of languages in that edition (6,809). So I looked at the Krauss article to see what it actually did say. The only thing I found that sounded similar to the above was on page 6: “10% are classed as ‘nearly extinct’ or ‘highly bilingual’, not warranting translation work.” I checked the 1988 edition and on page 741 is a chart of summary statistics which gives a count of 201 nearly extinct languages (or 3% of the then total of 6,170 languages). That edition of Ethnologue does not give a summary count for ‘highly bilingual’, but a companion publication does, and it says 247 or 4% (p. x). (Barbara F. Grimes, ed. 1988. Bible Translation Needs: Bulletin 1. Dallas, TX: Wycliffe bible Translators International.) That would give a total of 7%, so even the 10% that Krauss reports does not check out, and it is certainly not 17% (10% of the 60%).

In rereading Krauss, I note that he said, “the Grimeses themselves might agree that as many as 20% of the world 's languages are already moribund” (page 6). That appears to be an admission by Krauss that 20% was an upper limit on the number of language descriptions in the 1988 Ethnologue that could be construed as describing a moribund language. He was thus making the claim, without even giving any examples of cases in which Ethnologue had it wrong, that at least 30% of the languages were being described as vital when in fact they were moribund. That is a claim that could have been falsified by a third-party reviewer at the time by evaluating a small systematic sample of Ethnologue descriptions; it is unfortunate that no one did this so as to nip one-language-is-dying-every-two-weeks in the bud.

L162: “Assuming a global total of 6,500 known, currently spoken”.

L185: “Assuming 6,500 languages worldwide,”

L220: “Assuming a global total of about 6,500 languages,”

I wonder why the authors are asking us to assume a number that is well below what both Ethnologue and Glottolog are saying. Fig 2 shows that Ethnologue (following ISO 639) is documenting about 7,500 languages (both living and extinct). The exact number in the current edition is 7,618, and this is significantly lower than the number of languages in Glottolog. I’ve done some calculations on the CLDF download of Glottolog Database 5.1 and found that it has nearly 8,600 languoids at the Language level. Even with about 400 being deprecated in the Bookkeeping category, that would give a count of around 8,200 languages. One clue to the authors’ intent may be in the designation “currently spoken”, which could mean to exclude extinct languages, in which case the current number in Ethnologue is 7,170 and the corresponding number for Glottolog would be well above that. If we subtract the 346 languages classified as Dormant (with no remaining L1 users, but which some use as a language of heritage identity), then it goes 176 below 7,000—but the Glottolog number for that would still be well over 7,00. Depending on which categories of languages are meant to be included in the “assumed” global total, it seems that raising the “assumed” number of languages to 7,000 or 7,500 would be in order.

L166ff: Table 2, where 18% is reported as the Not Transmitted number for Ethnologue, attributed to Simons 2019.

I didn’t recognize the 18% figure, so I double checked the original paper. I did not find that 18% occurs within it. Figure 2 shows a histogram of the distribution of EGIDS values in the dataset and the text above it reports that EGIDS 7-8b comprise 14.4% and EGIDS 9-10 are 9.6%, which would give a total of 24% for languages that are no longer being transmitted.

L193. “consider the adjacent EGIDS category ‘Shifting,’ which includes languages with partially interrupted transmission.”

This sentence is describing EGIDS 6b and the correct label for that is “Threatened”. The label “Shifting” goes with EGIDS 7 and is used correctly above this in L183.

L236. “either as specific years (e.g., 2000) or broader ranges (e.g., the 2000s)”

We use the latter form with -s to mean sometime in the decade, but in the absence of our documentation, 2000s could also be interpreted as sometime in the century. Thus it would be better to use an example without that kind of ambiguity, with a specific year like 1994 and the matching decade rage of 1990s. For ranges broader that a decade we use phrases like “early 19th century”.

L245ff. Below et al report that “since 1960, … between 230 and 256 languages … have become dormant or extinct”

I can easily replicate this against the Ethnologue database. The Table of Languages in the Ethnologue Global Dataset lists the Year Last Speaker Died (YLSD) for every language that we report as having no remaining L1 speakers. The 29th edition dataset lists 534 languages with a YLSD of 1960 or greater. That works out to 534 /66 or 8.1 per year since 1960. That is slightly lower than the 9 in the 2019 paper, because the rate is rising and 9 is the rate in just the latest 25 year period.

L273. “Despite providing estimates that differ by roughly a factor of two,”

The first time I read the paper, I marked this is not being right, since 26 to 9 is a factor of three, and 26 to 4 is a factor of 6. The second time I read through the paper, I realized that you must mean the comparison between the Ethnologue estimate and the ELP, for which the factor of 2 is correct. So I am just alerting you to the potential misunderstanding, since the Krauss and Crystal estimate of 26 per year is the broader basis of comparison that is set in the opening sentence of the paper.

L278. remaining -> remainder

L293ff. “These figures should not be interpreted as a complete record of the world’s linguistic diversity at each point in time. In its early editions in particular, Ethnologue omitted many languages that were already known to linguists,”

I’ll just add a note to say that if you read the Introductions to the various editions, the early editions do not claim to be complete lists of languages; they aim only to be a growing list of “languages that do not have the Bible.” It is not until the 1970s that Introduction changes to say that the book aims to be comprehensive list of all known languages.

L301ff. “many languages remained entirely undocumented as recently as the 1960s and 1970s, the starting point for the extinction counts discussed above. As a result, estimates derived from these counts necessarily exclude the disappearance of a substantial number of languages and are therefore biased downward.”

Being added to the list of languages after 1960 does not affect the Ethnologue counts. The current list of 534 languages mentioned above that we believe to have fallen silent since 1960 makes no reference to when the language entered the inventory of known languages. In fact, it is likely that the majority have been added since 1960, since (as shown in Figure 2) Ethnologue listed only about 3,000 languages at that time. To this day, new languages are being added to the inventory when they are recognized by ISO 639, and if extinct, they are added with their estimated YLSD. Thus if the latter is 1960 or later, they will also be added to the count of languages that have gone extinct since 1960.

L 315ff. “Table 2. Distribution of languages by the reference year of speaker number estimates.”

The year of the population estimate is not a standard data point included in the Ethnologue Global Dataset, so I am guessing that this table has been generated from a scrape of the website. It was easy for me to go into the frozen database of the 27th edition and run a query to replicate Table 2, and the result is that the database is in significantly better shape than is reported in Table 2. First of all, we actually record the populations by language-in-country, rather than by language. Thus the population reported for a language is the sum of the estimates for all the countries it is recorded in. Table 2 says that it is based on populations for 5,627 living languages, but the total number of population estimates for L1 users in the 27th edition database was 11,392 (for that many languages-in-country). Only 149 of those were missing a year. (Instances in which the population is 0 were omitted from the count since they do not need to continue being updated in subsequent editions.) The Note under Table 2 says, “For nearly two thirds of these languages, the reference year is at least 15 years old, and for about one in six it is at least 25 years old,” but the query on the database finds that only 36.3% are at least 15 years old and that 11.5% are at least 25 years old. Here is what I get when trying to reproduce Table 2 with a SQL query that aggregates over all instances of L1 User population estimates in the 27th edition database:

Decade N Pct_of_Total

NULL 149 1.3

1950-59 2 0.0

1960-69 3 0.0

1970-79 72 0.6

1980-89 397 3.5

1990-99 845 7.4

2000-09 2820 24.8

2010-19 3105 27.3

2020-24 3999 35.1

L345. “Simons’ analysis points instead to a roughly stable rate between 1971–1995 and 1996–2018”

Presumably the “roughly stable” period is referencing the nearly horizontal segment in my Figure 4. However, Figure 5 shows what is behind that and it is not stability. In the Americas and Australia, the majority of languages were already past the point of doom so the rate of doom was in decline, whereas in the rest of the world were the majority of languages are vital, language doom was on the rise---so the downward trend in the parts of the world that are past the peak of language endangerment was counteracting the upward trend in the parts of the world where language endangerment is on the rise.

# Reviewer report: Review: What do we know about the language extinction rate? — R0/PR4

**DOI:** 10.1017/ext.2026.10019.pr4

**Authors:** Diane Nelson

**Affiliations:** School of Languages, Cultures and Societies, University of Leeds, United Kingdom of Great Britain and Northern Ireland

Dear Michaël and Harald, apologies for taking so long with the review. I really enjoyed reading this paper and I hope it gets published. I removed the “one language every two weeks” statistic from my own work a while ago but will now approach estimates of language loss with even more caution!

This article seeks to challenge a ubiquitous statistic about language extinction, namely that one language disappears every two weeks. In doing this the authors trace the linguistic myth back to its origins, clarifying its context; they review the available sources of evidence around language death, coming up with significantly less pessimistic numbers; and finally they offer suggestions for database design that would allow researchers to track the loss of the world’s languages in a more accurate way.

This paper makes a series of excellent points about the challenges in calculating rates of language loss and the paucity of accurate and current data. Their central argument is persuasive and empirically sound. Their schema on p. 9 is a useful and original way to conceptualise the gaps in the data in the current databases. The paper is well-written and coherent. Given the dangers of allowing an inaccurate statistic to promulgate in the media, research and education, the study is well-motivated and I believe it makes an important contribution to the field, for researchers, activists and policymakers alike. Prisms: Extinction is a suitable outlet for this paper and I recommend its publication subject to a few relatively minor editorial corrections as follows:

Minor editorial corrections:

p. 4, lines 55-6: ‘does a language die every two weeks? 2024’ Is this a citation to published work by Crystal or someone else? Nothing similar is listed in the references. If a source by that name is in the public domain, then this paper should cite it as closely related work with a similar aim.

p. 6, lines 156-7: I’m not sure if there’s a way to verify this, but I’m pretty sure that UNESCO’s World Atlas of Languages has been offline since longer than 2025, or at least the clickable map hasn’t worked for several years.

p. 6, lines 155-65 and p. 7 lines 173-81: for this paragraph and for the similar one on Ethnologue on the following page, it would be useful for the authors to reproduce the UNESCO vitality scale and EGIDS so that readers can get a sense of the authors’ criteria for inclusion in the set of languages equivalent to Krauss’s ‘moribund’ category. The readership of Prisms: Extinction are unlikely to share a background in linguistics and it would be useful as a reference point for them to see how these scales for formulated for languages.

In the same paragraphs, the authors use the figure of 6500 languages known as a baseline for their calculation of the number of languages no longer being transmitted. The use the same figure for Ethnologue. It would be good for them to justify their use of this number since we’re not told of the total number of languages in the UNESCO World Atlas, and Figure 2 on p. 10 seems to suggest that the Ethnologue database total is closer to 7500 for the 17th edition.

p. 7, lines 166-171: Table 1 should ideally be placed after the discussion of the relevant databases (p. 8). Something odd has happened to the citation parentheses in the legend.

p. 7, line 175: delete was, after EGIDS

p. 7, line 176: A bit more description of Fishman’s framework for characterising language shift would help the non-linguists grasp the argument here

p. 8, line 224: This section heading ‘A “more realistic approach”’ should be renamed; this section outlines several different approaches to calculating several statistics, and includes quite a few critiques of approaches rather than proposed solutions. It’s also not clear why the quotation marks are there.

p. 8, 230: ‘go extinct’ Somewhere in this paper, the authors should add a footnote (or maybe it should be included in the impact statement) to clarify that although they use the term throughout the paper, the term ‘extinct’ for languages is contentious, and is considered harmful by some Indigenous groups (Leonard 2011, Perley 2012). This is why databases are now using terms like dormant, sleeping and reawakening to describe languages with no living speakers. This will help situate the paper within the cross-disciplinary readership of Prisms: Extinction. Throughout the paper, I suggest the authors avoid expressions like “go extinct” in favour of “stop being spoken” or “pass out of daily use”.

p. 10, line 278: remaining > remainder

p. 11, line 315-319: Table 2 is a really interesting and useful set of data. I realise that the point here is about the likelihood of Ethnologue records being out of date and therefore underestimating rates of language loss, but it’s also important to note that only 5,627 entries out of 7,168 contain a reference year at all. That itself represents a big hole in Ethnologue’s dataset and it would be useful to point this out to the reader.

p. 12, line 354: I would suggest renaming this section, since its main aim is to propose an approach that might yield more accurate data. It should also be numbered (3).

p. 14 lines 424-434: these suggestions for better demographic information is a good idea, but I wonder if organisations like ELDP don’t already have guidance on this for their fieldworkers and researchers archiving language data? The authors might want to check this.

Wesley Y. Leonard, ‘Challenging “Extinction” through Modern Miami Language Practices’, American Indian Culture and Research Journal, 35.2 (2011), pp. 135–60.

Bernard C. Perley, ‘Zombie Linguistics: Experts, Endangered Languages and the Curse of Undead Voices’, Anthropological Forum, 22.2 (2012), pp. 133–49 (p. 134).

# Editor report: Recommendation: What do we know about the language extinction rate? — R0/PR5

**DOI:** 10.1017/ext.2026.10019.pr5

**Affiliations:** Durrell Institute of Conservation and Ecology, University of Kent, United Kingdom of Great Britain and Northern Ireland

This is a great paper and while I have never worked in this area it is a subject I have wanted to look at and therefore I found this manuscript very useful and accessible. As you can see the reviewers also enjoyed the manuscript and have a few minor corrections before it can be recommended that it be accepted. I look forward to seeing the revised manuscript and being able to accept it.

# Editor report: Decision: What do we know about the language extinction rate? — R0/PR6

**DOI:** 10.1017/ext.2026.10019.pr6

**Affiliations:** School of Natural Sciences, University of Tasmania, Australia

No accompanying comment.

# Editor report: Recommendation: What do we know about the language extinction rate? — R1/PR8

**DOI:** 10.1017/ext.2026.10019.pr8

**Affiliations:** Durrell Institute of Conservation and Ecology, University of Kent, United Kingdom of Great Britain and Northern Ireland

Thank you for considering all the points raised by the reviewers and for making corrections to the manuscript. I am now satisfied with the corrections and recommend it be accepted. I very much look forward to seeing it published and giving it as recommended reading to my students.

# Editor report: Decision: What do we know about the language extinction rate? — R1/PR9

**DOI:** 10.1017/ext.2026.10019.pr9

**Affiliations:** School of Natural Sciences, University of Tasmania, Australia

No accompanying comment.
